# Association of estimated glomerular filtration rate and urine albumin-to-creatinine ratio with incidence of cardiovascular diseases and mortality in chinese patients with type 2 diabetes mellitus – a population-based retrospective cohort study

**DOI:** 10.1186/s12882-017-0468-y

**Published:** 2017-02-02

**Authors:** Colman Siu Cheung Fung, Eric Yuk Fai Wan, Anca Ka Chun Chan, Cindy Lo Kuen Lam

**Affiliations:** Department of Family Medicine and Primary Care, the University of Hong Kong, 3/F Ap Lei Chau Clinic, 161 Main Street, Ap Lei Chau, Hong Kong

**Keywords:** Diabetes mellitus, Estimated glomerular filtration rate (eGFR), Urine albumin-to-creatinine ratio (UACR), Cardiovascular diseases, Mortality, Primary care

## Abstract

**Background:**

Estimated glomerular filtration rate (eGFR) and urine albumin-to-creatinine ratio (UACR) are renal markers associated with risks of cardiovascular diseases (CVD) and all-cause mortality in diabetic patients. This study aims to quantify such risks in Chinese diabetic patients based on eGFR and UACR.

**Methods:**

This was a territory-wide retrospective cohort study on primary care diabetic patients with documented eGFR and UACR but without baseline CVD in 2008/2009. They were followed up till 2013 on CVD events and mortality. Associations between eGFR/UACR and incidence of CVD/mortality were evaluated by multivariable Cox proportional models adjusted with socio-demographic and clinical characteristics.

**Results:**

The data of 66,311 patients who had valid baseline eGFR and UACR values were analysed. The risks of CVD events and mortality increased exponentially with the decrease in eGFR, with a hazard ratio (HR) increasing from 1.63 to 4.55 for CVD, and from 1.70 to 9.49 for mortality, associated with Stage 3 to 5 CKD, compared to Stage 1 CKD. UACR showed a positive linear association with CVD events and mortality. Microalbuminuria was associated with a HR of 1.58 and 2.08 for CVD and mortality in male (1.48 and 1.79 for female), respectively, compared to no microalbuminuria. Male patients with UACR 1–1.4 mg/mmol and eGFR ≥90 ml/min/1.73 m^2^ (60–89 ml/min/1.73 m^2^) had a HR of 1.25 (1.43) for CVD. Female patients with UACR 2.5–3.4 mg/ml and eGFR ≥90 ml/min/1.73 m^2^ (60–89 ml/min/1.73 m^2^) had a HR of 1.45 (1.65) for CVD.

**Conclusions:**

Risks of CVD events and mortality increased exponentially with eGFR drop, while UACR showed positive predictive linear relationships, and the risks started even in high-normal albuminuria. UACR-based HR was further modified according to eGFR level, with risk progressed with CKD stage. Combining eGFR and UACR level was more accurate in predicting risk of CVD/mortality. The findings call for more aggressive screening and intervention of microalbuminuria in diabetic patients.

## Background

Chronic kidney disease is one of the major complications in patients with Type 2 Diabetes Mellitus (T2DM). Renal impairment in diabetic patients can be manifested as a decrease in the estimated glomerular filtration rate (eGFR), the progression from microalbuminuria to macroalbuminuria to proteinuria, or both. Despite diabetic nephropathy is typically characterized by albuminuria (as commonly assessed by urine albumin-to-creatinine ratio, UACR), previous studies showed that albuminuria may be absent in some diabetic patients with an abnormal eGFR. Both low eGFR level and albuminuria had been shown to be independent poor prognostic factors for patients with diabetes [1–9]. UACR and eGFR were two commonly used indicators to assess renal function, and were found to be associated with cardiovascular and all-cause mortality [10]. There were studies trying to explore the relationship between eGFR and albuminuria on the prognosis of patients with diabetes mellitus (DM). A strong synergistic interaction between eGFR and albuminuria was found, in addition to the independent association of these parameters with mortality and progression to end-stage renal disease [11]. Studies in non-Chinese population had shown that eGFR and albuminuria were predictors of cardiovascular diseases (CVD) and mortality [1, 12–14]. However, there was limited information on whether eGFR and UACR have similar predictive values, if any, on CVD and mortality in Chinese patients with Type 2 DM, and the relationship between UACR and eGFR on clinical outcomes.

This study aims to explore the association between eGFR with both the risks of CVD events and all-cause mortality, and the risk modification by co-existing albuminuria. The association of albuminuria and CVD events and all-cause mortality would be quantified. The association between different levels of eGFR and albuminuria with risks of CVD events and all-cause mortality in patients with T2DM would also be identified.

Study objectives are to: [1] explore the relationship between eGFR and risks of CVD and all-cause mortality. [2] quantify the association between albuminuria and risks of CVD and all-cause mortality. [3] reveal the association between eGFR and albuminuria through identifying different hazard ratios (HR) on CVD events and all-cause mortality of patients with T2DM based on different levels of eGFR and albuminuria.

## Methods

### Study design

This is a territory-wide retrospective cohort study with Chinese subjects aged between 18 and 79. All subjects were clinically diagnosed with T2DM, with no prior CVD event and had DM management in one of the 74 General Out-Patient Clinics of the Hong Kong Hospital Authority (HA) across the whole territory. HA is the centralized organization that governs all public-sector hospitals and primary care clinics in Hong Kong and manages over half of all diabetic patients under primary care. Clinical data from 1 August 2008 to 31 December 2009 were collected from a territory-wide study for the evaluation of local diabetic programmes [15]. Through the administrative database of HA, clinical diagnosis of T2DM was identified by the International Classification of Primary Care-2 (ICPC-2) code of ‘T90’. Each patient was observed from their earliest record of eGFR/UACR, as the baseline date, to the following events whichever came first: the date of incidence of outcome event or all-cause mortality, or the last follow-up as censoring until 31 December 2013.

### Definitions

The Kidney Disease: Improving Global Outcomes (KDIGO) organization developed clinical practice guidelines in 2012 [16, 17] in which GFR Category 1 (or CKD Stage 1) was defined as eGFR ≥90 ml/min/1.73 m^2^, GFR Category 2 (or CKD Stage 2) as eGFR 60–89 ml/min/1.73 m^2^, Category G3 (including G3a and G3b, or CKD Stage 3) as eGFR 30–59 ml/min/1.73 m^2^, Category G4 (or CKD Stage 4) as eGFR <30 ml/min/1.73 m^2^, and GFR G5 (or CKD stage 5) as eGFR <15 ml/min/1.73 m^2^. The KDIGO also had a definition on albuminuria with A1 (normal to mildly increased) as ACR < 30 mg/g (or < 3 mg/mmol), A2 (moderately increased) as ACR 30–300 mg/g (or 3–30 mg/mmol), and A3 (severely increased) as > 300 mg/g (or > 30 mg/mmol). We adopted the definition of microalbuminuria and diabetic nephropathy in the local Hong Kong Reference Framework for Diabetes Care for Adults in Primary Care Settings, in which microalbuminuria was defined as UACR > 2.5 mg/mmol in men and > 3.5 mg/mmol in women, and UACR > 25 mg/mmol as diabetic nephropathy (or macroalbuminuria) [18].

### Cardiovascular diseases and mortality identification

Outcomes of interest included three events: 1) CVD event with one of the following subtype diagnoses: coronary heart disease (CHD), stroke, or heart failure, 2) all-cause mortality and 3) composite of CVD and all-cause mortality. Diagnosis of comorbidities was identified with the diagnosis coding system of ICPC-2 and International Classification of Diseases, Ninth Edition, Clinical Modification (ICD-9-CM). CHD including ischaemic heart disease, myocardial infarction, coronary death and sudden death was taken as ICPC-2 of K74 to K76 or ICD-9-CM of 410.x, 411.x to 414.x, 798.x. Heart failure was taken as ICPC-2 of K77 or ICD-9-CM of 428.x. Stroke including fatal and non-fatal was taken as ICPC-2 of K89 to K91 or ICD-9-CM of 430.x to 438.x.

### Baseline eGFR, UACR and other measurements

Clinical baseline eGFR and UACR readings in the patient records were extracted for analysis. The eGFR was calculated based on the creatinine level from blood test according to the abbreviated Modification of Diet in Renal Disease Study formula recalibrated for Chinese (eGFR in ml/min/1.73 m^2^ = 186 × [(serum creatinine in μmol/L) × 0.011] ^-1.154^ × (age)^-0.203^ × (0.742 if female) × 1.233, where 1.233 is the adjusted coefficient for Chinese [19]. Urine ACR was estimated based on spot urine sample for albumin to creatinine ratio. Baseline covariates included socio-demographics, clinical parameters, disease characteristics and treatment modalities of patients. Socio-demographics consisted of gender, age, smoking status and drinking habit. Clinical parameters were Haemoglobin A1c (HbA1c), body mass index (BMI), waist-to-hip ratio (WHR), systolic blood pressure (SBP), diastolic blood pressure (DBP), lipid profile (Low-density lipoprotein-cholesterol (LDL-C) and total cholesterol to high-density lipoprotein cholesterol ratio (TC/HDL-C ratio)) and triglyceride (TG). Disease characteristics composed of self-reported family history of DM, diagnosed hypertension and duration of DM. Hypertension was defined as clinical diagnosis with ICPC-2 code of “K86” or “K87”. Treatment modalities composed of usage of anti-hypertensive drug(s), oral anti-diabetic drug(s), insulin and lipid-lowering agent(s). All laboratory assays were performed in accredited laboratories by the College of American Pathologists of the Hong Kong Accreditation Service or the National Association of Testing Authorities in Australia.

### Data analysis

Missing data was handled by multiple imputation [20]. Each missing value was imputed five times by the chained equation method, equivalent to attain a relative efficiency of 95% [21, 22]. For each of the five imputed datasets, the same analysis was performed and the five sets of results were combined using Rubin’s rules [21].

All subjects were categorized in the following three ways: 1) as one of the five groups according to baseline eGFR value (≥90 ml/min/1.73 m^2^, 60–89 ml/min/1.73 m^2^, 30–59 ml/min/1.73 m^2^, 15–29 ml/min/1.73 m^2^ and <15 ml/min/1.73 m^2^); 2) as one of the eleven groups according to baseline UACR value (<0.5 mg/mmol, 0.5–0.9 mg/mmol, 1–1.4 mg/mmol, 1.5–1.9 mg/mmol, 2–2.4 mg/mmol, 2.5–3.4 mg/mmol, 3.5–4.9 mg/mmol, 5–9.9 mg/mmol, 10–24.9 mg/mmol, 25–34.9 mg/mmol and ≥35 mg/mmol); 3) as one of the 21 combinations of eGFR (≥90 ml/min/1.73 m^2^, 60–89 ml/min/1.73 m^2^ and <60 ml/min/1.73 m^2^) and UACR (<1 mg/mmol, 1–1.4 mg/mmol, 1.5–2.4 mg/mmol, 2.5–3.4 mg/mmol, 3.5–24.9 mg/mmol, 25–34.9 mg/mmol and ≥35 mg/mmol). All UACR groups were further stratified according to gender. Descriptive statistics were shown after multiple imputation for each subgroup of eGFR and UACR.

Differences in baseline characteristics between groups were assessed using ANOVA for continuous variables or chi-square test for categorical variables. Incidence rate was estimated by an exact 95% confidence interval (CI) based on a Poisson distribution [23]. Differences in incidences of CVD, all-cause mortality and composite of CVD and all-cause mortality between groups were tested by log-rank tests. The eGFR and UACR groups associated with incidences of CVD, all-cause mortality and composite of CVD and all-cause mortality were examined using multivariable Cox proportional hazards regressions, with adjustment of all baseline covariates. The interaction term between eGFR and UACR as the continuous variables was also tested. Proportional hazards assumption was checked by examining plots of the scaled Schoenfeld residuals against time for the covariates and the presence of multi-collinearity was assessed through variance inflation factor.

All significance tests were two-tailed and those with a *p-*value less than 0.05 were considered statistically significant. Statistical analysis was performed using STATA Version 13.0.

## Results

A total of 160,609 Chinese subjects with T2DM, eGFR or UACR measurements, aged ≥18, received their DM care in primary care clinics of HA from 1 August 2008 to 31 December 2010. With the exclusion of 13,457 patients with prior CVD event and 199 patients without follow-up after baseline, the remaining 146,953, 67,334 and 66,311 patients with valid record of eGFR, UACR and both, respectively, were included for analysis. Data completion rates for baseline factors were over 80%.

Table 1 displays the baseline characteristics for each eGFR group after multiple imputation. Relative to the lower eGFR groups, higher eGFR groups had 1) lower HbA1c, BMI, DBP and LDL-C; 2) a relatively longer duration of DM; and 3) a larger proportion of usage of insulin.

**Table 1:** Socio-demographic and clinical characteristics at baseline among estimated glomerular filtration rate groups

	Total (*N =* 146,953)	eGFR Group 1 (≥90 ml/min/1.73 m^2^) (*N =* 100,785)	eGFR Group 2 (60-89 ml/min/1.73 m^2^) (*N =* 38,537)	eGFR Group 3 (30–59 ml/min/1.73 m^2^) (*N =* 7,067)	eGFR Group 4 (15–29 ml/min/1.73 m^2^) (*N =* 431)	eGFR Group 5 (<15 ml/min/1.73 m^2^) (*N =* 133)	*P-*value
Socio-demographics							
Gender, (%, n)							<0.001
Female	54.8% (80,506)	53.3% (53,694)	56.4% (21,720)	66.6% (4,705)	70.1% (302)	63.9% (85)	
Male	45.2% (66,447)	46.7% (47,091)	43.6% (16,817)	33.4% (2,362)	29.9% (129)	36.1% (48)	
Age, years (mean ± SD)	63.91 ± 11.68	60.49 ± 10.78	70.37 ± 9.85	76.21 ± 9.02	79.28 ± 8.88	76.89 ± 11.25	<0.001
Smoking status, (%, n)							<0.001
Non-smoker	74.5% (109,484)	74.0% (74,541)	75.3% (29,013)	77.8% (5,495)	76.9% (331)	77.3% (103)	
Ex-smoker	15.0% (21,983)	14.2% (14,264)	16.8% (6,493)	16.1% (1,135)	16.8% (72)	14.1% (19)	
Current smoker	10.5% (15,486)	11.9% (11,980)	7.9% (3,031)	6.2% (436)	6.4% (27)	8.6% (11)	
Drinking habit, (%, n)							<0.001
Non-drinker	96.9% (142,369)	96.6% (97,320)	97.4% (37,554)	98.2% (6,943)	98.8% (426)	95.0% (126)	
Current drinker	3.1% (4,584)	3.4% (3,465)	2.6% (983)	1.8% (124)	1.2% (5)	5.0% (7)	
Clinical parameters							
HbA1c, % (mean ± SD)	7.31 ± 1.42	7.40 ± 1.49	7.14 ± 1.25	6.96 ± 1.23	6.63 ± 1.23	6.58 ± 1.55	<0.001
BMI, kg/m^2^ (mean ± SD)	25.51 ± 4.06	25.57 ± 4.27	25.43 ± 3.82	25.07 ± 4.39	24.86 ± 4.88	24.66 ± 4.25	<0.001
Waist hip ratio (mean ± SD)	0.93 ± 0.20	0.93 ± 0.20	0.94 ± 0.23	0.95 ± 0.19	0.96 ± 0.46	0.94 ± 0.09	<0.001
SBP, mmHg (mean ± SD)	136.69 ± 18.05	135.78 ± 17.70	138.58 ± 18.31	139.28 ± 20.12	137.99 ± 21.88	137.69 ± 22.07	<0.001
DBP, mmHg (mean ± SD)	75.57 ± 10.58	76.62 ± 10.34	73.83 ± 10.63	70.67 ± 10.82	67.90 ± 11.48	67.19 ± 12.59	<0.001
LDL-C, mmol/L (mean ± SD)	3.14 ± 0.88	3.14 ± 0.86	3.14 ± 0.91	3.08 ± 1.01	2.97 ± 0.95	2.98 ± 1.19	<0.001
TC/HDL-C ratio (mean ± SD)	4.40 ± 1.33	4.36 ± 1.34	4.46 ± 1.41	4.54 ± 1.47	4.54 ± 2.79	4.46 ± 2.92	<0.001
Triglyceride, mmol/L (mean ± SD)	1.69 ± 1.17	1.67 ± 1.21	1.74 ± 1.12	1.80 ± 1.13	1.72 ± 1.23	1.61 ± 1.51	<0.001
EGFR, ml/min/1.73 m^2^ (mean ± SD)	101.60 ± 44.27	114.74 ± 47.10	77.79 ± 8.04	50.34 ± 7.34	24.30 ± 4.05	10.59 ± 3.13	<0.001
UACR, mg/mmol (mean ± SD)	7.92 ± 40.51	5.32 ± 27.58	11.03 ± 49.02	25.51 ± 76.21	42.97 ± 111.42	25.47 ± 102.86	<0.001
Disease characteristics							
Family history of DM, (%, n)							<0.001
No	58.1% (85,324)	53.5% (53,924)	66.9% (25,774)	73.6% (5,201)	75.7% (326)	73.5% (98)	
Yes	41.9% (61,629)	46.5% (46,861)	33.1% (12,763)	26.4% (1,866)	24.3% (105)	26.5% (35)	
Diagnosed Hypertension, (%, n)							<0.001
No	33.6% (49,356)	40.7% (41,058)	19.1% (7,360)	12.1% (855)	13.2% (57)	19.5% (26)	
Yes	66.4% (97,597)	59.3% (59,727)	80.9% (31,177)	87.9% (6,212)	86.8% (374)	80.5% (107)	
Duration of DM, years (mean ± SD)	6.35 ± 7.08	5.74 ± 6.51	7.35 ± 7.50	9.34 ± 9.50	10.14 ± 10.37	9.22 ± 11.69	<0.001
Treatment modalities							
Anti-hypertensive drugs, (%, n)							<0.001
No	32.5% (47,768)	39.0% (39,329)	19.3% (7,432)	12.9% (911)	14.6% (63)	24.8% (33)	
Yes	67.5% (99,185)	61.0% (61,456)	80.7% (31,105)	87.1% (6,156)	85.4% (368)	75.2% (100)	
Oral anti-diabetic drug used, (%, n)							<0.001
No	30.3% (44,531)	30.7% (30,931)	30.1% (11,613)	25.2% (1,781)	34.3% (148)	43.6% (58)	
Yes	69.7% (102,422)	69.3% (69,854)	69.9% (26,924)	74.8% (5,286)	65.7% (283)	56.4% (75)	
Insulin used, (%, n)							<0.001
No	99.1% (145,570)	99.1% (99,922)	99.1% (38,171)	98.3% (6,947)	95.4% (411)	89.5% (119)	
Yes	0.9% (1,383)	0.9% (863)	0.9% (366)	1.7% (120)	4.6% (20)	10.5% (14)	
Lipid-lowering agents, (%, n)							<0.001
No	91.1% (133,868)	91.7% (92,388)	89.8% (34,599)	90.1% (6,364)	91.6% (395)	91.7% (122)	
Yes	8.9% (13,085)	8.3% (8,397)	10.2% (3,938)	9.9% (703)	8.4% (36)	8.3% (11)	

*CVD* Cardiovascular Diseases, *DM* Diabetes Mellitus, *BMI* Body Mass Index, *HbA1c* Haemoglobin A1c, *SBP* Systolic Blood Pressure, *DBP* Diastolic Blood Pressure, *LDL-C* Low-density Lipoprotein-Cholesterol, *TC* Total Cholesterol, *HDL-C* High-density Lipoprotein-Cholesterol, *UACR* Urine Albumin/Creatinine Ratio, *eGFR* estimated Glomerular Filtration Rate

**Fig. 1:**
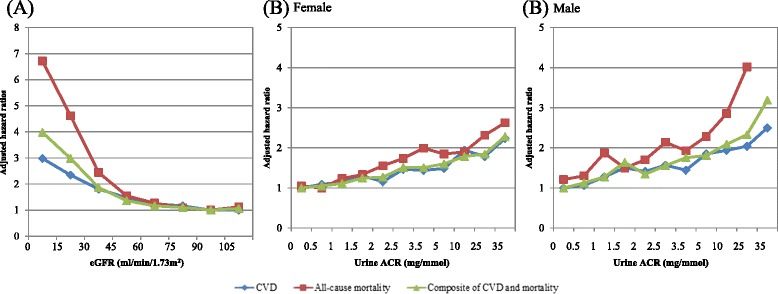
Adjusted hazard ratios for incidence of cardiovasular diseases (CVD), all-cause mortality and composite of CVD and mortality by updated mean (**a**) estimated glomerular filtration rate (eGFR) and (**b**) urine albumin/creatinine (UACR) ratio by multivariable Cox proportional hazards regression

Tables 2, 3 and 4 and Fig. 1 show the number, unadjusted incidence rates, and adjusted HR of the three outcome events for each eGFR and UACR group. During a median follow-up period of 14.5–51.5 months, incidence rates of composite of CVD and all-cause mortality were between 22.1 and 401.3 per 1,000 person-years among all eGFR groups. Similarly, incidence rate of composite of CVD and all-cause mortality were between 12.4 and 65.5 per 1,000 person-years among all UACR groups during a median follow-up period of 41.5–47.5 months for female; and between 16.1 and 79.5 per 1,000 person-years during a median follow-up period of 41.5–46.5 months for male. The risks of CVD events and mortality increased exponentially with the decrease in eGFR, with HR raised from 1.63 to 4.55 (from CKD Stage 3 to 5) for CVD and from 1.70 to 9.49 (from CKD Stage 3 to 5) for all-cause mortality, compared with CKD Stage 1. Urine ACR showed a positive linear association with CVD and mortality. Microalbuminuria had HR of 1.58 (male) and 1.48 (female) for CVD; and 2.08 (male) and 1.79 (female) for mortality. HR rose to 2.57 and 4.36 respectively for male, and 2.40 and 3.07 respectively for female, if progressed to frank albuminuria.

**Table 2:** Number and incidence rate of CVD, all-cause mortality and composite of CVD and all-cause mortality among estimated glomerular filtration rate (eGFR) groups by multivariable Cox proportional hazard regression

	eGFR Group 1 (≥90 ml/min/1.73 m^2^) (*N =* 100,785)	eGFR Group 2 (60–89 ml/min/1.73 m^2^) (*N =* 38,537)	eGFR Group 3 (30–59 ml/min/1.73 m^2^) (*N =* 7,067)	eGFR Group 4 (15–29 ml/min/1.73 m^2^) (*N =* 431)	eGFR Group 5 (<15 ml/min/1.73 m^2^) (*N =* 133)
CVD					
Cumulative Cases with Event	5,552	4,426	1,404	134	38
Cumulative Incidence Rate	5.5%	11.5%	19.9%	31.1%	28.6%
Person-years	4,925,567	1,823,869	301,183	12,069	2,871
Medium follow-up (Months)	51.5	50.5	47.5	24.5	14.5
Incidence Rate (95% CI) ^a^	13.53 (13.18,13.89)	29.12 (28.28,29.99)	55.94 (53.09,58.94)	133.24 (112.49,157.82)	158.86 (115.59,218.32)
Hazard Ratio (95% CI)	Reference group	1.17* (1.12,1.22)	1.63* (1.52,1.73)	3.17* (2.66,3.78)	4.55* (3.29,6.31)
All-cause mortality					
Cumulative Cases with Event	4,416	3,512	1,546	252	90
Cumulative Incidence Rate	4.4%	9.1%	21.9%	58.5%	67.7%
Person-years	5,041,933	1,910,744	325,858	13,944	3,166
Medium follow-up (Months)	51.5	51.5	49.5	35.5	16.5
Incidence Rate (95% CI)^a^	10.51 (10.20,10.82)	22.06 (21.34,22.80)	56.93 (54.16,59.84)	216.88 (191.69,245.38)	341.18 (277.50,419.47)
Hazard Ratio (95% CI)	Reference group	0.99 (0.95,1.04)	1.70* (1.59,1.81)	5.19* (4.55,5.93)	9.49* (7.68,11.73)
Composite of CVD and mortality					
Cumulative Cases with Event	9,062	6,817	2,374	287	96
Cumulative Incidence Rate	9.0%	17.7%	33.6%	66.6%	72.2%
Person-years	4,925,567	1,823,869	301,183	12,069	2,871
Medium follow-up (Months)	51.5	50.5	47.5	24.5	14.5
Incidence Rate (95% CI)^a^	22.08 (21.63,22.54)	44.85 (43.80,45.93)	94.59 (90.86,98.47)	285.37 (254.19,320.37)	401.32 (328.56,490.20)
Hazard Ratio (95% CI)	Reference group	1.03 (1.00,1.07)	1.51* (1.44,1.58)	4.09* (3.63,4.62)	7.08* (5.78,8.68)

Hazard ratios were adjusted for age, gender, smoking status, drinking habit, body mass index, waist-to-hip ratio, glycated hemoglobin A1c, systolic and diastolic blood pressure, low-density lipoprotein-cholesterol, total cholesterol to high-density lipoprotein cholesterol ratio, triglyceride, urine albumin/creatinine ratio, self-reported duration of diabetes mellitus, family history of diabetes mellitus, diagnosed hypertension, the usage of anti-hypertensive drugs, oral anti-diabetic drugs, insulin and lipid-lowering agents at baseline

*CVD* Cardiovascular Diseases, *eGFR* estimated Glomerular Filtration Rate

*Significant difference (*P <* 0.05) by multivariable Cox proportional hazards regression

^a^Incidence rate (cases/1000 person-years) with 95%CI based on Poisson distribution

**Table 3:** Number and incidence rate of CVD, all-cause mortality and composite of CVD and all-cause mortality among urine albumin/creatinine ratio (UACR) groups (male) by multivariable Cox proportional hazard regression

	UACR Group 1 (<0.5 mg/mmol) (*N =* 5,821)	UACR Group 2 (0.5–0.9 mg/mmol) (*N =* 8,796)	UACR Group 3 (1–1.4 mg/mmol) (*N =* 3,846)	UACR Group 4 (1.5–1.9 mg/mmol) (*N =* 2,183)	UACR Group 5 (2–2.4 mg/mmol) (*N =* 1,474)	UACR Group 6 (2.5–3.4 mg/mmol) (*N =* 1,906)	UACR Group 7 (3.5–4.9 mg/mmol) (*N =* 1,563)	UACR Group 8 (5–9.9 mg/mmol) (*N =* 2,308)	UACR Group 9 (10–24.9 mg/mmol) (*N =* 1,765)	UACR Group 10 (25–34.9 mg/mmol) (*N =* 406)	UACR Group 11 (≥35 mg/mmol) (*N =* 1,021)
CVD											
Cumulative Cases with Event	242	417	245	167	112	159	123	248	206	50	154
Cumulative Incidence Rate	4.2%	4.7%	6.4%	7.7%	7.6%	8.3%	7.9%	10.7%	11.7%	12.3%	15.1%
Person-years	268,568	399,457	172,506	95,953	65,849	84,092	67,881	99,719	74,531	16,277	39,981
Medium follow-up (Months)	46.5	45.5	44.5	44.5	45.5	44.5	43.5	43.5	43.5	41.5	41.5
Incidence Rate (95% CI)^a^	10.81 (9.53,12.26)	12.53 (11.38,13.79)	17.04 (15.04,19.32)	20.89 (17.95,24.31)	20.41 (16.96,24.56)	22.69 (19.42,26.51)	21.74 (18.22,25.95)	29.84 (26.35,33.80)	33.17 (28.93,38.02)	36.86 (27.94,48.64)	46.22 (39.47,54.13)
Hazard Ratio (95% CI)	Reference group	1.07 (0.91,1.26)	1.30* (1.09,1.56)	1.57* (1.28,1.91)	1.42* (1.13,1.78)	1.58* (1.29,1.94)	1.48* (1.19,1.84)	1.94* (1.62,2.33)	2.00* (1.65,2.42)	2.21* (1.63,3.01)	2.57* (2.08,3.17)
All-cause mortality											
Cumulative Cases with Event	138	277	156	126	79	112	115	165	155	45	160
Cumulative Incidence Rate	2.4%	3.1%	4.1%	5.8%	5.4%	5.9%	7.4%	7.1%	8.8%	11.1%	15.7%
Person-years	273,362	407,234	176,630	99,035	68,040	86,781	70,042	103,939	78,012	17,049	42,556
Medium follow-up (Months)	46.5	45.5	45.5	45.5	45.5	44.5	44.5	44.5	44.5	42.5	42.5
Incidence Rate (95% CI)^a^	6.06 (5.13,7.16)	8.16 (7.26,9.18)	10.60 (9.06,12.40)	15.27 (12.82,18.18)	13.93 (11.18,17.37)	15.49 (12.87,18.64)	19.70 (16.41,23.65)	19.05 (16.35,22.19)	23.84 (20.37,27.91)	31.67 (23.65,42.42)	45.12 (38.64,52.68)
Hazard Ratio (95% CI)	Reference group	1.23* (1.00,1.51)	1.41* (1.12,1.77)	1.93* (1.51,2.46)	1.53* (1.16,2.02)	1.79* (1.39,2.31)	2.29* (1.78,2.94)	2.08* (1.65,2.62)	2.41* (1.90,3.05)	3.26* (2.32,4.59)	4.36* (3.43,5.53)
Composite of CVD and mortality											
Cumulative Cases with Event	361	648	359	264	165	239	217	353	315	77	265
Cumulative Incidence Rate	6.2%	7.4%	9.3%	12.1%	11.2%	12.5%	13.9%	15.3%	17.8%	19.0%	26.0%
Person-years	268,568	399,457	172,506	95,953	65,849	84,092	67,881	99,719	74,531	16,277	39,981
Medium follow-up (Months)	46.5	45.5	44.5	44.5	45.5	44.5	43.5	43.5	43.5	41.5	41.5
Incidence Rate (95% CI)^a^	16.13 (14.55,17.88)	19.47 (18.02,21.02)	24.97 (22.52,27.69)	33.02 (29.26,37.25)	30.07 (25.81,35.03)	34.11 (30.04,38.72)	38.36 (33.58,43.82)	42.48 (38.27,47.15)	50.72 (45.41,56.64)	56.77 (45.40,70.97)	79.54 (70.52,89.72)
Hazard Ratio (95% CI)	Reference group	1.14 (1.00,1.29)	1.32* (1.14,1.53)	1.70* (1.45,1.99)	1.37* (1.14,1.65)	1.59* (1.34,1.87)	1.85* (1.56,2.19)	1.88* (1.62,2.18)	2.16* (1.85,2.52)	2.64* (2.06,3.39)	3.41* (2.89,4.03)

Hazard ratios were adjusted for age, gender, smoking status, drinking habit, body mass index, waist-to-hip ratio, glycated hemoglobin A1c, systolic and diastolic blood pressure, low-density lipoprotein-cholesterol, total cholesterol to high-density lipoprotein cholesterol ratio, triglyceride, estimated Glomerular Filtration Rate, self-reported duration of diabetes mellitus, family history of diabetes mellitus, diagnosed hypertension, the usage of anti-hypertensive drugs, oral anti-diabetic drugs, insulin and lipid-lowering agents at baseline

*CVD* Cardiovascular Diseases, *UACR* Albumin/Creatinine Ratio

*Significant difference (*P <* 0.05) by multivariable Cox proportional hazards regression

^a^Incidence rate (cases/1000 person-years) with 95%CI based on Poisson distribution

**Table 4:** Number and incidence rate of CVD, all-cause mortality and composite of CVD and all-cause mortality among urine albumin/creatinine ratio (UACR) groups (female) by multivariable Cox proportional hazard regression

	UACR Group 1 (<0.5 mg/mmol) (*N =* 3,277)	UACR Group 2 (0.5–0.9 mg/mmol) (*N =* 9,836)	UACR Group 3 (1–1.4 mg/mmol) (*N =* 5,714)	UACR Group 4 (1.5–1.9 mg/mmol) (*N =* 3,245)	UACR Group 5 (2–2.4 mg/mmol) (*N =* 2,210)	UACR Group 6 (2.5–3.4 mg/mmol) (*N =* 2,694)	UACR Group 7 (3.5–4.9 mg/mmol) (*N =* 2,155)	UACR Group 8 (5–9.9 mg/mmol) (*N =* 2,965)	UACR Group 9 (10–24.9 mg/mmol) (*N =* 2,306)	UACR Group 10 (25–34.9 mg/mmol) (*N =* 502)	UACR Group 11 (≥35 mg/mmol) (*N =* 1,341)
CVD											
Cumulative Cases with Event	115	414	295	195	128	195	161	257	268	59	215
Cumulative Incidence Rate	3.5%	4.2%	5.2%	6.0%	5.8%	7.2%	7.5%	8.7%	11.6%	11.8%	16.0%
Person-years	154,111	455,441	260,056	148,173	100,254	120,471	95,349	130,004	100,133	21,349	54,205
Medium follow-up (Months)	47.5	46.5	45.5	45.5	45.5	44.5	44.5	44.5	44.5	43.5	41.5
Incidence Rate (95% CI)^a^	8.95 (7.46,10.75)	10.91 (9.91,12.01)	13.61 (12.14,15.26)	15.79 (13.72,18.17)	15.32 (12.88,18.22)	19.42 (16.88,22.35)	20.26 (17.36,23.65)	23.72 (20.99,26.81)	32.12 (28.49,36.20)	33.16 (25.69,42.80)	47.60 (41.64,54.40)
Hazard Ratio (95% CI)	Reference group	1.14 (0.93,1.40)	1.20 (0.97,1.49)	1.34* (1.06,1.69)	1.21 (0.94,1.56)	1.51* (1.20,1.90)	1.48* (1.17,1.89)	1.58* (1.27,1.98)	2.00* (1.60,2.49)	1.84* (1.34,2.53)	2.40* (1.90,3.03)
All-cause mortality											
Cumulative Cases with Event	52	184	151	102	83	114	113	163	146	47	151
Cumulative Incidence Rate	1.6%	1.9%	2.6%	3.1%	3.8%	4.2%	5.2%	5.5%	6.3%	9.4%	11.3%
Person-years	156,248	463,725	265,206	151,359	102,518	123,314	98,097	134,594	104,628	22,326	57,656
Medium follow-up (Months)	48.5	46.5	45.5	46.5	45.5	45.5	45.5	45.5	45.5	44.5	43.5
Incidence Rate (95% CI)^a^	3.99 (3.04,5.24)	4.76 (4.12,5.50)	6.83 (5.83,8.01)	8.09 (6.66,9.82)	9.72 (7.83,12.05)	11.09 (9.23,13.33)	13.82 (11.50,16.62)	14.53 (12.46,16.94)	16.75 (14.24,19.69)	25.26 (18.98,33.62)	31.43 (26.79,36.86)
Hazard Ratio (95% CI)	Reference group	1.08 (0.80,1.48)	1.27 (0.92,1.74)	1.43* (1.02,1.99)	1.60* (1.13,2.26)	1.77* (1.27,2.47)	2.08* (1.49,2.90)	1.97* (1.43,2.70)	2.07* (1.50,2.85)	2.60* (1.74,3.88)	3.07* (2.22,4.25)
Composite of CVD and mortality											
Cumulative Cases with Event	159	560	395	265	193	271	237	374	340	88	296
Cumulative Incidence Rate	4.9%	5.7%	6.9%	8.2%	8.7%	10.1%	11.0%	12.6%	14.7%	17.5%	22.1%
Person-years	154,111	455,441	260,056	148,173	100,254	120,471	95,349	130,004	100,133	21,349	54,205
Medium follow-up (Months)	47.5	46.5	45.5	45.5	45.5	44.5	44.5	44.5	44.5	43.5	41.5
Incidence Rate (95% CI)^a^	12.38 (10.60,14.46)	14.75 (13.58,16.03)	18.23 (16.52,20.12)	21.46 (19.03,24.21)	23.10 (20.06,26.60)	26.99 (23.96,30.41)	29.83 (26.26,33.88)	34.52 (31.19,38.20)	40.75 (36.64,45.32)	49.46 (40.14,60.96)	65.53 (58.47,73.44)
Hazard Ratio (95% CI)	Reference group	1.12 (0.94,1.33)	1.17 (0.97,1.41)	1.31* (1.08,1.60)	1.34* (1.09,1.66)	1.58* (1.29,1.92)	1.57* (1.28,1.93)	1.73* (1.43,2.08)	1.89* (1.56,2.29)	2.00* (1.54,2.60)	2.54* (2.08,3.09)

Hazard ratios were adjusted for age, gender, smoking status, drinking habit, body mass index, waist-to-hip ratio, glycated hemoglobin A1c, systolic and diastolic blood pressure, low-density lipoprotein-cholesterol, total cholesterol to high-density lipoprotein cholesterol ratio, triglyceride, estimated Glomerular Filtration Rate, self-reported duration of diabetes mellitus, family history of diabetes mellitus, diagnosed hypertension, the usage of anti-hypertensive drugs, oral anti-diabetic drugs, insulin and lipid-lowering agents at baseline

*CVD* Cardiovascular Diseases, *UACR* Urine Albumin/Creatinine Ratio

*Significant difference (*P <* 0.05) by multivariable Cox proportional hazards regression

^a^Incidence rate (cases/1000 person-years) with 95%CI based on Poisson distribution

**Fig. 2:**
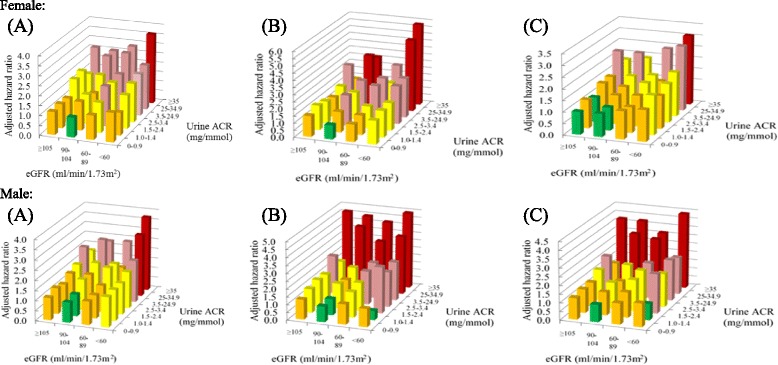
Adjusted hazard ratios for incidence of **a** cardiovasular diseases, **b** all-cause mortality and **c** a composite of cardiovasular diseases and all-cause mortality by updated estimated glomerular filtration rate (eGFR) and urine albumin/creatinine ratio (UACR) compared to the reference group with eGFR 90–104 ml/min/1.73 m^2^ and UACR 0–0.9 mg/mmol. Hazard ratios were adjusted for age, gender, smoking status, drinking habit, body mass index, waist-to-hip ratio, glycated hemoglobin A1c, systolic and diastolic blood pressure, low-density lipoprotein-cholesterol, total cholesterol to high-density lipoprotein cholesterol ratio, triglyceride, self-reported duration of diabetes mellitus, family history of diabetes mellitus, diagnosed hypertension, the usage of anti-hypertensive drugs, oral anti-diabetic drugs, insulin and lipid-lowering agents at baseline

The adjusted HR of CVD, all-cause mortality and composite of CVD and all-cause mortality of different combinations of eGFR and UACR are summarized in Fig. 2. Male patients with UACR 1–1.4 mg/mmol and eGFR ≥90 ml/min/1.73 m^2^ (60–89 ml/min/1.73 m^2^) had a HR of 1.25 (1.43) for CVD. Male patients with UACR 1.5–2.4 mg/mmol and eGFR ≥90 ml/min/1.73 m^2^ (60–89 ml/min/1.73 m^2^) had a HR of 1.54 (1.56) for all-cause mortality, which raised further to 2.51 if eGFR was <60 ml/min/1.73 m^2^. Female patients with UACR 2.5–3.4 mg/ml and eGFR ≥90 ml/min/1.73 m^2^ (60–89 ml/min/1.73 m^2^) had a HR of 1.45 (1.65) for CVD. Female patients with UACR 2.5–3.4 mg/mmol and eGFR 60–89 ml/min/1.73 m^2^ (<60 ml/min/1.73 m^2^) had a HR of 2.03 (2.17) for all-cause mortality. The optimal eGFR and UACR for preventing CVD, all-cause mortality and composite of CVD and all-cause mortality was ≥90 ml/min/1.73 m^2^ for eGFR and <1 mg/mmol for UACR. The interaction effect between eGFR and UACR for each outcome event was statistically significant. The risk of developing CVD, all-cause mortality and composite of CVD and all-cause mortality increased significantly in other combinations of eGFR and UACR.

## Discussion

This is the first study to examine in details the kidney function, in terms of eGFR and albuminuria, and its association with CVD and mortality in Chinese patients with T2DM at the primary care setting by stratifying patients into different categories based on different levels of eGFR and UACR. The risks of CVD, all-cause mortality, and the composite of the two dropped exponentially with increasing eGFR. The number of patients with CKD stage 5 and 4 was very small and hence they were not separately categorized out during analysis. Patients with CKD Stage 2 were shown to have a significant increase in cardiovascular risk (HR 1.17), and the risk elevated further as CKD stage progressed (HR 4.55 for CKD Stage 5). Patients with CKD stage 3 were found to have significant increase in mortality risk (HR 1.70), which escalated further as CKD stage progressed (HR 9.49 for CKD stage 5). For patients at CKD stage 3, they had significant increase in risks of CVD (HR 1.63), all-cause mortality (HR 1.70), and composite of the two (HR 1.51). In female, even with normal albuminuria, patients with eGFR < 90 ml/min/1.73 m^2^ had a significantly higher risk of CVD than those with eGFR ≥ 90 ml/min/1.73 m^2^, but not for all-cause mortality. This pattern was not prominent in male. This is not in agreement with another study that suggested a suboptimal eGFR was a strong predictor for major CVD in diabetic patients with normoalbuminuria [24]. Such gender difference needs further exploration.

Hong Kong had launched a local reference framework for Diabetes Care for Adults in Primary Care Settings Hong Kong in 2010 in which the cut-offs of microalbuminuria and macroalbuminuria were 2.5 mg/mmol and 25 mg/mmol for male, and 3.5 mg/mmol and 35 mg/mmol for female respectively [18]. Nevertheless, our study showed the risks of CVD and all-cause mortality were indeed elevated even in low urine albumin level of 0.5–0.9 mg/mmo1. This supports the trend of using the terms “moderately increased albuminuria” and “severely increased albuminuria” (to replace “microalbuminuria” and “macroalbuminuria”, respectively) [16, 17] as the cardiovascular risk did not show an all-or-none relationship but a positive linear relationship with urine albumin level. Male patients with UACR as low as 0.5–0.9 mg/mmol were already found to have increased risk for all-cause mortality (HR 1.23), whereas a male diabetic patient with UACR as low as 1–1.4 mg/mmol were found to have a significant enhanced risk for CVD (HR 1.30). Male patients with microalbuminuria (UACR ≥2.5 mg/mmol) had a HR between 1.58 and 1.79 for outcomes of interest. The HR elevated further (between 2.21 to 3.26) if they developed macroalbuminuria. A similar pattern was found in female diabetic patients. The HR of low eGFR and high albuminuria on CVD and all-cause mortality found in our study were comparable to other studies [14, 25, 26].

The amount of albumin leak through the glomerulus is directly linked to the degree of glomerular damage. The more advanced the glomerular injury, the less chance to recover to a normal function. UACR generally runs a positive linear association with CVD, all-cause-mortality, and a composite of the two. This supports that patients with microalbuminuria should be screened and intervened early. The definition of microalbuminuria and macroalbuminuria varies [18, 27], and their cut-offs are more like the conceptual terms for easy reference. It is the amount of albumin that leaks and passes into urine that matters to a clinician. The link of microalbuminuria with cardiovascular events may be attributed to the endothelial dysfunction in diabetic patients [5]. Presence of albuminuria is sensitive to detecting CVD as even with a UACR as low as 1–1.4 mg/mmol, a significantly increased risk of CVD or all-cause mortality was found in both men and women. Indeed high-normal albuminuria was also found to be associated with an increased CVD risk [2, 3, 28–31]. The differences of the cut-offs for micro/macroalbuminuria in male and female did not show much differences in the outcome as the linear relationship showed the more albumin in urine, the higher risk a patient had. Presence of frank albuminuria double or triple the CVD and mortality risk in both male and female diabetic patients. Converting a diabetic patient with macroalbuminuria back to microalbuminuria halved the risk. Such association between albuminuria and cardiovascular or mortality risk was found similar to that in non-Chinese [1, 26, 32].

Despite that the amount of albumin leaked into urine may be transient or fluctuant due to many factors such as fever or exercise [33], urine ACR seems to have more impact than eGFR on the clinical practice. Firstly, it was theoretically reversible and modifiable. Secondly, testing for albuminuria was considered to be more informative than testing eGFR alone. After leakage through the glomerulus, albumin is not only excreted into urine, but is also reabsorbed by tubules. Hence, presence of albuminuria suggests damages in both glomerular and tubules, in contrast to worsening of eGFR where only glomerulus damage is suggested [34]. Thirdly, UACR but not serum creatinine was found to have a significant association with HbA1c in diabetic patients with both good and poor control of diabetes [35]. UACR was found to have a significant positive correlation with HbA1c >8% and < 8%, but serum creatinine was only significantly associated in those with HbA1c >8% [35]. UACR was a more specific marker for diabetic nephropathy and hence a closer relationship with DM. The analysis had suggested that “moderately increased albuminuria” over time is an important risk factor for CVD and early cardiovascular mortality [1, 5, 6, 36–39]. Nevertheless, various studies suggested that CKD had an independent association with cardiovascular events in diabetic patients [39–41].

Albuminuria control appeared to have less significance in patients with profound renal impairment as regardless how good the UACR control in patients with decreased eGFR was, their risk of cardiovascular events and all-cause mortality remained high. Tighter albuminuria control in patients with relatively preserved renal function (higher eGFR) had greater significance and positive impact on the prognosis in terms of cardiovascular risk and mortality. For patients with renal impairment with elevated serum creatinine (lower eGFR), the reduction in the amount of creatinine passed out into urine may over-estimate the UACR and it was not truly reflective of the severity of albuminuria. Although the benefit of screening UACR is comparatively less in patients with eGFR <60 ml/min/1.73 m^2^ when compared to patients with eGFR ≥60 ml/min/1.73 m^2^, knowing their UACR can provide clinicians a more complete picture, particularly when the renal impairment of the patient is caused by comorbidities other than diabetic nephropathy. Our study showed that UACR based HR could be further modified according to eGFR levels, with risk progressed with stage of CKD, and thus a more patient-centred HR can be assigned to an individual patient base on the eGFR and UACR level. With the readiness of converting frank albuminuria to microalbuminuria or even normoalbuminuria, a female diabetic patient with baseline eGFR <60 ml/min/1.73 m^2^ could have her HR of CVD decreased from 3.23 (albuminuria) to 1.99 (microalbuminuria) to further down (normoalbuminuria). Similarly, a male diabetic patient with baseline eGFR <60 ml/min/1.73 m^2^ could have his HR of CVD decreased from 2.71 (albuminuria) to 2.10 (microalbuminuria) to further down (normoalbuminuria). On the other hand, for patients with acceptable eGFR and started to develop mildly increased albuminuria, timely intervention like tight control of DM and the use of angiotensin-converting-enzyme inhibitors (ACEI) or angiotensin receptor blockers (ARB) can prevent or postpone the progression to moderately or severely increased albuminuria and the subsequent development of diabetic nephropathy, which in turn helps to maintain a satisfactory eGFR level. Controlling the UACR can also delay the rise of HR towards cardiovascular events and all-cause mortality, not to mention the renal complications which are not the focus of this article.

### Strengths and limitations of this study

One of the strengths of this study was that the sample size was large enough to represent the Chinese diabetic population in Hong Kong. In addition, relevant baseline covariates such as laboratory results, disease characteristics and treatment modalities were accessed through the HA’s computerised administrative database which provided reliable results. Furthermore, multiple imputations were used to handle missing data to overcome bias in the results.

On the other hand, several limitations were identified. Firstly, the analysis did not consider some lifestyle interventions such as regular exercise and diet modification, which may be potential contributors to CVD risk. However, baseline covariates such as duration of T2DM, BMI, WHR, HbA1c, BP, and lipid, in some sense, can reflect the intensity of disease severity and lifestyle modification. Secondly, a positive relationship between eGFR/UACR and risks of CVD and all-cause mortality was identified. However, this pattern of association may not be guaranteed in other Chinese populations and is subjected to temporal changes and modifications. Thirdly, the incidence of outcome events was dependent on the clinical diagnosis coding by ICPC-2 and ICD-9-CM codes and documentation in the database, which may subject to misclassification bias. Fourthly, although all the laboratories within the HA were certified, there may be possibilities of between laboratory drift regarding the results obtained from different laboratories within the HA across the whole territory. Lastly, the long-term effects of eGFR/UACR on risk of CVD and all-cause mortality are yet to be confirmed. Further longitudinal studies with a minimum follow-up period of 10 years will provide more evidence on the long-term association between eGFR/UACR and incidence of CVD and mortality.

## Conclusions

Risks of CVD events and all-cause mortality increased exponentially with eGFR drop, while UACR showed positive predictive linear relationships, and the risks started even in Chinese T2DM patients with high-normal albuminuria. UACR-based HR was further modified according to the eGFR levels, with risk progressed with CKD stage. Combining eGFR and UACR level was more accurate in predicting risk of CVD and all-cause mortality. Serum creatinine (eGFR) and urinary ACR should be regularly monitored in diabetic patients. Early intervention to halt or even reverse the progression reduces the risk of CVD and all-cause mortality. The findings call for more aggressive screening and intervention of microalbuminuria in diabetic patients.

## Abbreviations

ACEI: Angiotensin-converting enzyme inhibitors
ARB: Angiotensin receptor blockers
BMI: Body mass index
CHD: Coronary heart disease
CI: Confidence interval
CVD: Cardiovascular diseases
DBP: Diastolic blood pressure
DM: Diabetes mellitus
eGFR: Estimated glomerular filtration rate
HA: Hong Kong Hospital Authority
HbA1c: Haemoglobin A1c
HR: Hazard ratio
ICD-9-CM: International classification of diseases, ninth edition, clinical modification
ICPC-2: International classification of primary care-2
LDL-C: Low-density lipoprotein-cholesterol
SBP: Systolic blood pressure
SD: Standard deviation
T2DM: Type 2 diabetes mellitus
TC: total cholesterol
TC/HDL-C ratio: high-density-lipoprotein cholesterol ratio
TG: triglyceride
UACR: urine albumin-to-creatinine ratio
WHR: Waist to Hip ratio
